# Foot and Mouth Disease in Livestock and Reduced Cryptosporidiosis in Humans, England and Wales

**DOI:** 10.3201/eid0901.020512

**Published:** 2003-01

**Authors:** William J. Smerdon, Tom Nichols, Rachel M. Chalmers, Hilary Heine, Mark Reacher

**Affiliations:** * Public Health Laboratory Service–Communicable Disease Surveillance Centre, London, England; † Cryptosporidium Reference Unit, Singleton Hospital, Sgeti, Swansea, Wales

**Keywords:** Foot and mouth disease, livestock, Cryptosporidium, cryptosporidiosis, zoonoses, disease reservoirs, epidemiology, water, water treatment, research

## Abstract

During the 2001 epidemic of foot and mouth disease (FMD) in livestock in England and Wales, we discovered a corresponding decrease in laboratory reports of cryptosporidiosis in humans. Using a regression model of laboratory reports of cryptosporidiosis, we found an estimated 35% (95% confidence interval [CI] 20% to 47%) reduction in reports during the weeks spanning the period from the first and last cases of FMD. The largest reduction occurred in northwest England, where the estimated decrease was 63% (95% CI 31% to 80%). Genotyping a subgroup of human isolates suggested that the proportion of *Cryptosporidium* genotype 2 strain (animal and human) was lower during the weeks of the FMD epidemic in 2001 compared with the same weeks in 2000. Our observations are consistent with livestock making a substantial contribution to *Cryptosporidium* infection in humans in England and Wales; our findings have implications for agriculture, visitors to rural areas, water companies, and regulators.

*Cryptosporidium* is a genus of enteric parasites, a leading cause of infectious diarrhea in humans and livestock. Infection is accompanied by fecal shedding of large numbers of highly infectious and environmentally persistent oocysts (*1*). Transmission occurs through the fecal-oral route in animal-to-human or human-to-human contact, by recreational exposure to contaminated water or land, or by consumption of contaminated water and food. Infection is also frequently associated with travel to high incidence countries (*1*). In England and Wales, most isolates are characterized as genotype 1 (which infects only humans) and genotype 2 (which infects both livestock and humans) (*2*).

*Cryptosporidium* oocysts have also been recognized as a continuing challenge to water treatment during the last 20 years. Because *Cryptosporidium* organisms tend to become widely distributed in surface waters and are resistant to chlorination, if coagulation and filtration are inadequate in public water supplies, the contaminated water can cause large outbreaks (*1*,*3*,*4*). *Cryptosporidium* in water supplies was studied by three expert committees commissioned by U.K. departments of health and environment in the 1990s, which made recommendations on improving management of slurry, on human hygiene relating to livestock, and on best practices in water treatment and outbreak investigation (*5*–*7*). Most cases of cryptosporidiosis, however, are not associated with recognized outbreaks and the sources of these infections remain uncertain (*1*,*7*).

Human cryptosporidiosis outbreaks in the U.K. are recognized as being bimodal, peaking in the spring and fall (*1*). Spring peaks vary by year and location and have been attributed to lambing, calving, and the application of slurry, combined with high rainfall, leading to run-off from agricultural land into surface water and drinking water catchments (*1*). Autumn peaks have been attributed to persons’ summer travel to countries with higher incidence (*1*).

Surveillance of human enteric infection in England and Wales (which encompass 89% of the U.K. population [*8*]) is conducted by the voluntary reporting of positive laboratory test results from individual case-patients and outbreak summaries to the Public Health Laboratory Service–Communicable Disease Surveillance Centre (PHLS–CDSC) in Colindale, London. A computer database of individual laboratory reports was established in 1975; a database of outbreak summaries was established in 1992 (*9*,*10*). England and Wales have 229 microbiology laboratories, of which 47 are public health laboratories (*11*,*12*).

In 2001, in all regions of the U.K., an epidemic of foot and mouth disease (FMD) in livestock occurred (*13*) (Table 1). The following measures taken to control the epidemic: excluding visitors from the countryside, extensive culling of affected herds and flocks of farm animals, and limiting the movement of animals for trade and to and from pastures in affected areas (*13*). These measures likely reduced the direct and indirect exposure to livestock of the overall population of England and Wales. Therefore, we examined laboratory reports of human infection with *Cryptosporidium* for evidence of changes that may have occurred during the period of the FMD epidemic.

**Table 1:** Key events during the foot-and-mouth-disease epidemic in livestock, United Kingdom, 2001^a^

Dates	Cumulative cases	Event
**2001**		
19 February	0	FMD case suspected at an abattoir in Essex, southeast England.
20 February	1	Index case confirmed.
21 February	2	Animal movements banned within infected area. Ban on moving susceptible animals and nontreated animal products from the U.K. imposed by the European Commission.
23 February	6	Case confirmed in Northumberland, northeast England. Environment Agency and Ministry of Agriculture, Fisheries and Food issue joint statement that disposal of animal carcasses produced by culling constitutes an emergency under the terms of the Environmental Protection Act 1990.
25 February	7	Case confirmed in Devon, southwest England.
27 February	16	Special rights to close footpaths and rights of way outside infected areas granted to local government. First case in Wales (Anglesey).
28 February	24	First case in Cumbria, northwest England.
1 March	31	First case in Scotland (Dumfries and Galloway).
2 March	38	Animals intended for the human consumption permitted to be moved under license.
6 March	80	Environment Agency announces disposal hierarchy, placing rendering and incineration first.
15 March	250	Policy of culling sheep within 3 km of an infected premise announced by Minister of Agriculture.
20 March	394	Prime Minister initiates daily interdepartmental meetings chaired by Ministry of Agriculture, Fisheries and Foods.
23 March	514	First meeting of Cabinet Office Briefing Room, chaired by the Prime Minister. Government Chief Scientific Officer proposes a 24-h infected premises/48-h contiguous cull policy. 101 Logistics Brigade of the Army deployed at Ministry of Agriculture, Fisheries and Food headquarters.
26 March	644	First mass burial of animal carcasses at Great Orton, northeast England.
30 March	829	Largest number of new cases (50) reported in a single day.
15 April	1,320	14% of footpaths open.
7 May	1,563	Last carcasses buried at Great Orton. Last day of incineration of carcasses. Backlog of animals awaiting disposal cleared.
8 June	1,714	Prime Minister announces new Department for Environment, Food and Rural Affairs replacing Ministry of Agriculture, Fisheries and Food.
22 June	1,773	Department for Environment, Food and Rural Affairs announces intention to revoke most footpath closures.
30 September	2,026	Last confirmed case of foot and mouth disease in Cumbria, northwest England.
28 November	2,026	Last foot and mouth disease–infected area designations lifted from parts of Cumbria, northwest England, north Yorkshire, and County Durham, northeast England.
7 December	2,026	Guidance to lift remaining footpath restrictions issued.
**2002**		
14 January		Northumberland, northeast England, last county declared to be foot and mouth disease–free.
22 January		U.K. regains international foot and mouth disease–free status, clearing way to resume normal trade in animals and animal products.
21 June		National Audit Office report published “The 2001 Outbreak of Foot and Mouth Disease.” Six million animals slaughtered. Direct cost to public sector, 3 billion pounds (U.S. $4.7 billion). Cost to private sector, 5 billion pounds (U.S. $7.9 billion), mostly in the tourism sector. Up to 100,000 animals slaughtered and disposed of each day.

^a^Source, National Audit Office, U.K. (13).

## Methods

From the national laboratory database, we downloaded laboratory reports to PHLS–CDSC of *Cryptosporidium* oocysts identified in fecal smears with dates for obtaining specimens between January 1, 1991, and December 31, 2001. The download was performed on June 20, 2002, to ensure that all data for 2001 were complete.

The data were aggregated into counts by week the specimen was obtained . To make all weeks exactly 7 days long, we excluded reports with specimen dates on December 31 of every year and on December 30 of every leap year. Intervals between date of illness onset, specimen date, and reporting date were reviewed to assess consistency of reporting over time.

The exposure interval for the FMD epidemic was defined as weeks 8–39 of 2001, which corresponded with the first FMD case on February 20 and last case on September 30 (*13*) (Table 1). We plotted the series of *Cryptosporidium* reports for England and Wales over time and reviewed data from Wales and each region in England individually.

The weekly counts of reports were used as the dependent variable in a negative, binomial regression model. Explanatory variables were region, season (weeks 1–7, weeks 8–39, or weeks 40–52), year (1991–2001), FMD interval (weeks 8–39 in 2001), and a binary variable for a batch reporting error in 1995. We used negative binomial regression rather than Poisson regression because the variance of the count was not approximately equal to the mean of the count (*14*). An estimate of the reduction in the reports of cryptosporidiosis during the FMD interval was obtained from this model; this estimate was derived from the ratio of the mean count within weeks 8–39 in 2001 to the mean count during the same interval in all other years, adjusted for all other explanatory variables. For each year considered, the mean counts A, B, C, and D were summarized in a 2 X 2 table (Table 2). The rate ratio for the FMD interval = (A/B)/(C/D). A similar model was used to estimate a rate ratio for the FMD interval in each region separately and to estimate rate ratios for weeks 8–39 in 1991, 1992, and all other years.

**Table 2:** Mean counts of reports for weeks and years of foot-and-mouth-disease epidemic and weeks and years of non–foot-and-mouth-disease epidemic used to calculate risk ratios

	Yr 2001	Remaining yrs 1991–2000
Wks 8–39	A	B
Wks 1–7 and 40–52	C	D

Fecal specimens positive for *Cryptosporidium* species received by the PHLS Cryptosporidium Reference Unit in Swansea with dates of specimen between January 1, 2000, and December 31, 2001, were genotyped by using polymerase chain reaction and restriction fragment length polymorphism analysis of a region of the *Cryptosporidium* oocyst wall protein gene (*15*). The proportion of isolates of genotype 2 was examined. Monthly rainfall data for England and Wales were also examined for trends during the surveillance period.

## Results

The data set comprised 51,322 reports of *Crytosporidium*. We concluded that an outlying count of 387 reports with a specimen date of October 6, 1995, was a batch-reporting error of cases from a large waterborne outbreak in Torbay, in southwest England, which occurred during July–September 1995 (*9*). Annual reporting rates varied from 6.7–11/100,000 for England and Wales as a whole. Rates were generally <5/100,000 in London; in several recent years, the highest rates occurred in northwest England (Table 3).

**Table 3:** Annual rate of reported *Cryptosporidium* species per 100,000 population, England and Wales, 1991–2001^a^

Region in England	1991	1992	1993	1994	1995	1996	1997	1998	1999	2000	2001
Eastern	7.8	9.4	9.3	6.6	12.3	5.0	11.3	5.1	7.5	9.1	8.2
London	7.8	3.6	2.6	2.7	3.6	2.4	2.3	1.9	2.9	3.8	2.3
Northwest	11.7	17.6	12.9	10.6	11.7	10.1	15.8	11.9	19.8	20.9	7.5
Northern and Yorkshire	10.2	13.0	11.4	12.6	12.1	10.4	8.2	7.9	9.6	8.2	6.9
Southeast	11.5	6.8	8.2	8.8	10.2	5.5	6.9	5.9	6.6	8.7	6.7
Southwest	15.3	14.2	12.1	12.7	22.0	8.9	10.2	10.2	12.7	14.7	8.7
Trent	9.5	9.6	8.9	10.5	11.2	7.0	9.1	7.8	10.1	14.1	7.1
West Midlands	6.5	7.6	8.6	4.9	7.6	6.4	5.7	5.6	8.6	10.3	7.3
Wales	12.1	11.3	12.0	11.5	12.5	7.9	7.2	9.3	11.5	12.1	8.3
Total (England and Wales)	10.2	10.1	9.2	8.7	11.0	6.9	8.4	7.0	9.6	11.0	6.7

^a^Mid-year population estimates from the Office for National Statistics, U.K.

**Figure 1:**
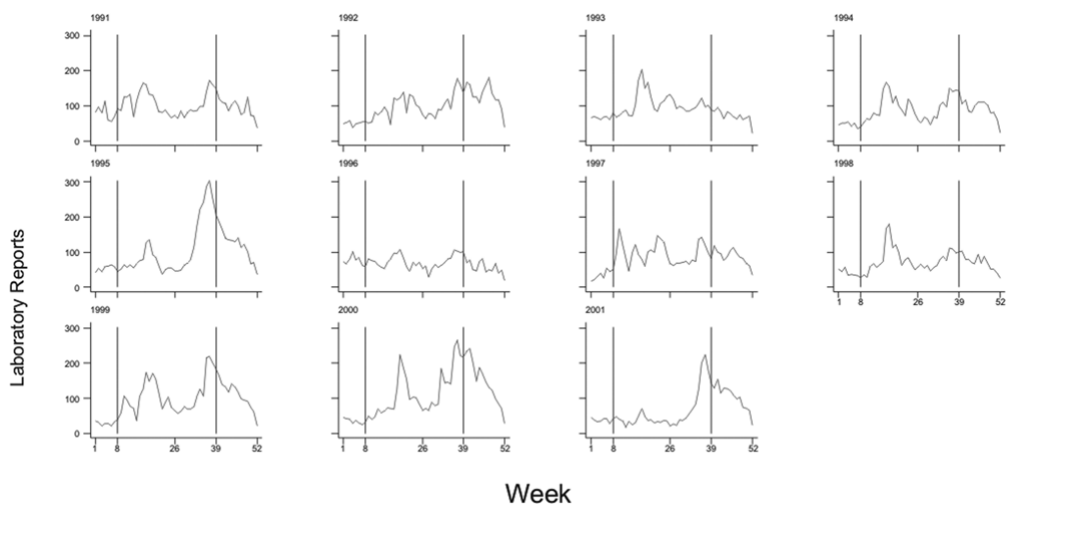
Laboratory reports of *Cryptosporidium* species to Public Health Laboratory Service – Communicable Disease Surveillance Centre, by specimen week, England and Wales, 1991–2001. Reporting artifact on October 6, 1995 not shown.

The timing of clearly identifiable spring and autumn peaks of *Cryptosporidium* laboratory reports was fairly consistent from year to year (Figure 1). Weeks 8–39 of each year included all spring peaks and the first half of all autumn peaks. However, spring and autumn peaks were not always clearly identifiable in each year. The year 1993 was notable for the absence of an autumn peak; in 1996 and 1997, identifying either a spring or autumn peak was difficult (Figure 1). In 2001, weekly counts remained lower than in previous years until approximately week 35, when an autumn peak comparable to previous years was observed (Figure 1).

By modeling counts, a significant decrease in overall seasonal and yearly associations was noted only in 1992 and in 2001 (Table 4). Weeks 8–39 in 1992 showed an estimated 22% decrease, whereas weeks 8–39 in 2001, corresponding to the FMD epidemic, showed an estimated 35% (95% confidence interval [CI] 20% to 47%) decrease in England and Wales as a whole (p=0.001). We estimated that the FMD epidemic interval was associated with some reduction of human cryptosporidiosis in all regions of England and in Wales, but the largest association was in northwest England, which showed decrease of approximately 63% (95% CI 31% to 80%) (p=0.001) (Table 5).

**Table 4:** Rate ratios for weeks 8–39 of each year adjusted for yearly and seasonal effects, England and Wales, 1991–2001

Yr	Rate ratio	95% confidence intervals	p value
1991	1.10	0.89 to 1.35	0.38
1992	0.78	0.64 to 0.96	0.02
1993	1.27	1.03 to 1.57	0.02
1994	1.10	0.89 to 1.35	0.38
1995	0.97	0.79 to 1.20	0.81
1996	1.02	0.83 to 1.26	0.82
1997	1.20	0.97 to 1.47	0.10
1998	1.11	0.90 to 1.38	0.32
1999	1.07	0.87 to 1.31	0.55
2000	0.93	0.76 to 1.14	0.47
2001	0.65	0.53 to 0.80	0.001

**Table 5:** Rate ratios associated with foot-and-mouth-disease epidemic, England and Wales, 2000

Region	Rate ratio	95% confidence intervals	p value
Northwest	0.37	0.20 to 0.69	0.001
Eastern	0.57	0.31 to 1.06	0.08
Northern and Yorkshire	0.60	0.32 to 1.11	0.10
Southeast	0.66	0.36 to 1.21	0.17
London	0.67	0.35 to 1.31	0.24
Southwest	0.72	0.39 to 1.34	0.30
Trent	0.90	0.48 to 1.68	0.74
West Midlands	0.90	0.48 to 1.69	0.75
Wales	0.70	0.37 to 1.33	0.28
Total (England and Wales)	0.65	0.53 to 0.80	0.001

**Figure 2:**
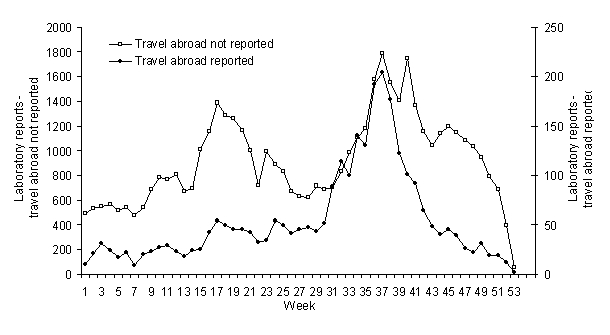
Laboratory reports of *Cryptosporidium* species to Public Health Laboratory Service–Communicable Disease Surveillance Centre, by specimen week, including reports with and without foreign travel, England and Wales, 1991–2001. FMD, foot and mouth disease.

The age distribution for persons with reported cryptosporidiosis for weeks 8–39 in 2001 was similar for the same time of year in each of the preceding 10 years. A history of foreign travel preceding diagnosis was given in 5% of reports and showed a single autumn peak in weeks 31–41 of each year, while those reports from case-patients without a history of foreign travel showed both spring and autumn peaks (Figure 2).

**Figure 3:**
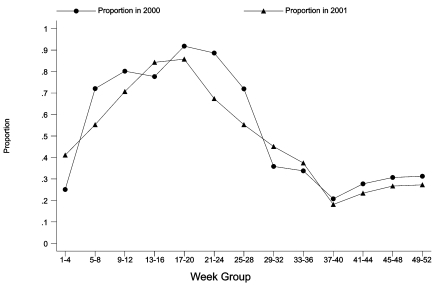
Laboratory isolates of *Cryptosporidium* species, proportion of genotype 2, by specimen week, England and Wales, 2000 and 2001.

Genotyping results were available for approximately half of the reported cases of cryptosporidiosis made to PHLS–CDSC in 2000 and 2001. When specimens from persons with a history of recent foreign travel were excluded, the proportion of genotype 2 cases was generally higher in the first half of both years (Figure 3). In weeks 21–24 of 2001 (May 21–June 17), the proportion of genotype 2 cases was significantly lower than for the same weeks of 2000 (Figure 3). In weeks 8–39 as a whole, the proportion of genotype 2 cases was significantly lower in 2001 than in 2000 (49%, 338/696 in 2001; 63%, 977/1,558 in 2000; p<0.00005).

Mean rainfall in England and Wales between January and June (to approximately week 26) was <60 mm in 1992 and 1996 (Table 6): in these years, the spring peak in *Cryptosporidium* reporting was also below normal (Figure 1). In contrast, rainfall in the first 6 months of 2001 was above the 1961–1990 average and showed a pattern more like that that observed in 1998, 1999, and 2000: in these years, a spring peak in *Cryptosporidium* reporting was not conspicuous (Figure 1; Table 6).

**Table 6:** Monthly precipitation values (mm), England and Wales, 1991–2001^a^

Month	Approx wks	Yr											
		1991	1992	1993	1994	1995	1996	1997	1998	1999	2000	2001	1961–90 mean
Jan	1–4	97.5	48.7	115.3	131	162.6	65.9	16.4	121.2	127.7	46.5	84.4	91.0
Feb	5–8	64.3	44.8	13.8	85.2	114.8	83.3	115.9	20.1	49.1	95.1	105.3	65.0
Mar	9–13	74.3	82.2	26.6	94.0	70.6	43.2	30.7	88.0	69.6	32.7	107.5	74.0
Apr	14–17	70.9	75.9	94.8	76.3	28.1	51.0	24.6	132.6	76.3	142.6	100.0	61.0
May	18–21	13.6	51.4	89.3	71.1	48.5	58.4	72.8	35.4	55.5	98.0	42.1	65.0
Jun	22–26	103.0	38.0	68.6	36.1	20.2	29.6	136.7	119.8	89.2	43.0	44.4	65.0
Jan–June mean	1–26	70.6	56.8	68.1	82.3	74.1	55.2	66.2	86.2	77.9	76.3	80.6	70.2
Jul	27–30	70.7	89.7	88.6	45.0	37.6	43.6	45.9	56.5	26.1	63.8	73.2	62.0
Aug	31–35	27.8	134.6	54.4	75.7	9.1	79.9	104.0	47.2	115.8	65.9	86.3	77.0
Sep	36–39	64.9	96.9	119.6	106.2	123.3	34.0	34.3	102.1	120.6	132.6	82.9	78.0
Oct	40–43	72.1	90.5	94.4	103.5	52.0	87.8	72.3	154.7	86.5	188.0	135.4	87.0
Nov	44–48	93.4	148.5	75.9	87.9	82.8	134.3	122.1	88.8	67.3	182.1	65.1	92.0
Dec	49–52	49.3	78.6	172.0	138.0	91.1	55.5	108.5	96.8	142.4	137.2	43.5	95.0
Jul–Dec mean	27–52	63.0	106.5	100.8	92.7	66.0	72.5	81.2	91.0	93.1	128.3	81.1	81.8
Annual mean	1–52	66.8	81.7	84.4	87.5	70.1	63.9	73.7	88.6	85.5	102.3	80.8	76.0

^a^Including the 1961–1990 mean. Source, University of East Anglia (*16*).

## Discussion

*Cryptosporidium* reports from England and Wales decreased substantially during the FMD epidemic in livestock in 2001, with a marked attenuation of reports in the first half of the year. Modeling counts of *Cryptosporidium* reports showed that the observed decrease was unlikely to be explained by seasonal and yearly associations. In none of the previous 10 years were deviations from overall yearly and seasonal associations as large as those estimated for weeks 8–39 in 2001.

For a number of reasons, this decrease cannot be attributed to errors in transmission and entry of reports into the PHLS–CDSC database. Counts returned to normal levels by about week 35 of 2001, followed by an autumn peak comparable in size to that in most previous years. A similar pattern of decrease was not apparent for *Salmonella*, *Campylobacter*, or *Giardia* reports, which were received and stored in a manner similar to those for *Cryptosporidium* (PHLS data). Lag times between onset of illness, specimen collection, and reporting dates were stable throughout the data set, except for the single batch-reporting artifact in 1995 (for which adjustment was made in the regression analysis). No change or disruption in reporting methods or data storage was known to have occurred over the study period.

Genotyping of specimens from 2000 and 2001 showed that most submitted were of genotype 2 (livestock and human strain) in the first half of each year, whereas most were of genotype 1 (human-only strain) in the second half of each year. During the FMD epidemic interval, the proportion of genotype 2 isolates tested was lower than that for the same time of year in 2000.

The presence of an autumn peak in case-patients reported to have recently traveled abroad, coincident with an autumn peak in case-patients not known to have recently traveled abroad, is consistent with substantial underreporting of travel abroad and the association of the autumn peak with such travel. Conversely, the absence of a spring peak among case-patients with reported recent travel abroad suggests that the spring peak is predominantly due to exposures occurring within the U.K.

The absence of clear spring peaks in human *Cryptosporidium* reports in 1992 and 1996 may have been related to below average rainfall in England and Wales in the first 6 months of these years. However, the rainfall levels in the first 6 months of 2001 were similar to those in 1998, 1999, and 2000, years in which spring peaks were conspicuous. Therefore, no strong evidence suggests that the exceptionally low number of reports observed during the FMD epidemic interval could be explained by below average rainfall.

The low reporting rate for *Cryptosporidium* in London may be explained by the historic shortage of public health laboratories in the capital. Public health laboratories, which provide approximately half of all laboratory reports to PHLS–CDSC, examine all fecal specimens for *Cryptosporidium* oocysts and report all positive results to PHLS–CDSC (*10*). Recent surveys indicate that 20% of laboratories in the northwest region of England and Wales and 40% of laboratories in the east and southeast regions of England continue to use varied criteria to select a subset of submitted fecal specimens for examination for *Cryptosporidium* oocysts, and may not always report positive test results (*17*,*18*): similar variation may be expected in other regions. However, we have no evidence to suggest that these laboratories systematically changed their practices during the study period. The recent comparatively high reporting rates of *Cryptosporidium* infections in the northwest region cannot be entirely explained by the fact that a higher proportion of laboratories now have policies requiring the examination of all fecal specimens for *Cryptosporidium* oocysts and the reporting of all positive results. The northwest region of England has experienced comparatively frequent confirmed and suspected waterborne outbreaks of cryptosporidiosis (*18*); therefore, a genuinely higher incidence is a more likely explanation. Throughout England and Wales, a substantial decrease in reports was observed, coinciding with the start of the FMD epidemic in 2001, followed by a return to normal levels by about week 35. A systematic change in testing and reporting by over 200 laboratories does not explain this observation.

On the whole, our results suggest that a decrease in genotype 2 *Cryptosporidium* infection in humans was associated with a decrease in human exposure to reservoirs of infection in livestock in England and Wales during the FMD epidemic interval. That the FMD epidemic interval was associated with a decrease in all English regions and Wales, including London, may have been because visitors from throughout England and Wales had decreased access to affected regions.

That contamination of water supplies was decreased through removal of livestock from drinking water catchments by slaughter or containment elsewhere is also plausible. The FMD epidemic was estimated to have had the largest effect in northwest England, which is consistent with the particularly large change in animal husbandry and livestock numbers associated with the FMD epidemic in this region. Livestock fecal contamination of an unfiltered surface water reservoir may have decreased in the English Lake District, which serves approximately one-third of the population of the northwest region with drinking water (*19*).

Water companies in England and Wales have been required to conduct risk assessments of their water sources for *Cryptosporidium* and to undertake real-time monitoring of treated water for oocysts at high-risk works since April 1, 2000 (*20*). However, introduction of this regulation was not associated with a decrease in *Cryptosporidium* reporting between April 1, 2000, and the beginning of the FMD epidemic on February 20, 2001.

The surveillance patterns observed suggest that exposure to livestock and their excreta may contribute a substantial fraction of human cryptosporidiosis in England and Wales. Our observations support continued concern over the presence of *Cryptosporidium* oocysts in public water supplies, especially in northwest England, and suggest that policies and the economics for the management of water catchments and water treatment in England and Wales, especially the northwest region, require further review.

The impact of the FMD epidemic in livestock on *Cryptosporidium* infection in humans can be characterized as complex. Long-lasting changes to farming practices and restructuring of rural economies occurred and will continue. Additionally, water companies continue to improve the microbiologic safety of public water supplies, supported by strict legal limits for *Cryptosporidium* oocyst concentrations in treated water. Whether the decline in *Cryptosporidium* reporting coincident with the FMD epidemic will be sustained in future years will be interesting to observe.

Changes in livestock-mediated exposure to *Cryptosporidium* would not correspond precisely with the interval between the first and last confirmed cases of FMD. A delay was expected between the start of the FMD epidemic in livestock and a change in livestock-mediated *Cryptosporidium* exposure in humans and its consequent detection by the national laboratory surveillance system. Key components of this delay include the incubation period of *Cryptosporidium* in humans; the amount of time before seeking medical attention, and the time required for giving a fecal test, examining the specimen, and reporting and entering positive test results into the national database. We expected the degree of change in livestock-mediated *Cryptosporidium* exposure to vary by time and place because of variation in livestock densities, the intensity of animal culling, and differences in the containment of animals from traditional pastures between different areas of the U.K. Nonetheless, the coincidence between the FMD epidemic and decline in human cryptosporidiosis is striking and suggests that the FMD epidemic in livestock has changed the ecology between humans, livestock, and *Cryptosporidium* in England and Wales.

Further studies to define the contribution of key components of the FMD epidemic on human *Cryptosporidium* infection may be of value in appropriate geographic areas such as northwest England. Such studies could include modeling the independent effect of changes in livestock densities, farm access, and rural access and adjusting for water supply to residences, changes in water treatment, and rainfall.
